# Spinach-based RNA mimicking GFP in plant cells

**DOI:** 10.1007/s10142-022-00835-x

**Published:** 2022-03-10

**Authors:** Zhiming Yu, Yue Wang, Fengling Mei, Haiting Yan, Zhenhui Jin, Pengcheng Zhang, Xian Zhang, Mahmut Tör, Stephen Jackson, Nongnong Shi, Yiguo Hong

**Affiliations:** 1 Research Centre for Plant RNA Signaling, College of Life and Environmental Sciences, Hangzhou Normal University, Hangzhou, 311121 China; 2 School of Science and the Environment, University of Worcester, Worcester, WR2 6AJ UK; 3 School of Life Sciences, University of Warwick, Coventry, CV4 7AL UK

**Keywords:** Onion epidermal cell, RNA aptamer, RNA fluorescence, *Spinach*-RMG

## Abstract

Spinach RNA-mimicking GFP (S-RMG) has been successfully used to monitor cellular RNAs including microRNAs in bacterium, yeast, and human cells. However, S-RMG has not been established in plants. In this study, we found that like bacterial, yeast, and human cellular tRNAs, plant tRNAs such as tRNA^Lys^ can protect and/or stabilize the Spinach RNA aptamer interaction with the fluorophore DFHBI enabling detectable levels of green fluorescence to be emitted. The tRNA^Lys^-Spinach-tRNA^Lys^, once delivered into “chloroplast-free” onion epidermal cells can emit strong green fluorescence in the presence of DFHBI. Our results demonstrate for the first time that Spinach-based RNA visualization has the potential for in vivo monitoring of RNAs in plant cells.

**Supplementary Information:**

The online version contains supplementary material available at 10.1007/s10142-022-00835-x.

## Introduction

RNAs primarily act as messengers to convey genetic information from DNA to protein. However, the functionalities of RNAs are much broader. Increasing evidence has demonstrated that RNAs can be potent regulators modulating gene expression at the transcriptional, post-transcriptional, and translational levels. In plants, cellular mRNAs, small interfering RNA, microRNAs, and pathogenic viral and viroid RNAs can move from cell to cell through plasmodesmata and spread to distal tissues via the phloem superhighway (Uddin and Kim 2013; Thieme et al. 2015; Liu and Chen 2018). Some of these mobile RNAs function as intra- and intercellular as well as systemic signals to control plant defense, growth and development, and responses to environmental stresses (Jackson and Hong 2012; Liu and Chen 2018; Zhang et al. 2019). For instance, *BEL5* mRNA moves from the leaf to stolon tip to promote potato tuber formation and development (Banerjee et al. 2006), and mobile *Flowering Locus T* (*FT*) mRNAs regulate flowering in *Arabidopsis* (Li et al. 2009; 2011; Lu et al. 2012; Luo et al. 2018). A short segment of the *FT* RNA that confers mobility has also been exploited to enhance heritable gene editing (Ellison et al. 2020). Furthermore, many RNAs are able to move across hetero-graft scions between different plants (Notaguchi et al. 2015) or ecotypes (Thieme et al. 2015), between parasitic plant and its hosts in a bidirectional manner, or even between plants and fungi (Uddin and Kim 2013; Kim et al. 2014). These emerging frontiers in plant RNA biology require novel technologies to study and visualize RNAs in plant cells.

RNAs can be visualized in living cells using molecular beacons (MBs), RNA-binding labeled proteins (RBLPs), and RNA aptamer-based approaches (Tutucci et al. 2018). MBs involve a specific probe that perfectly complements the target RNA in homogeneous solutions. RBLPs, such as MS2, PUM-HD, hnRNPA1, λN22, Cas9, and Cas13a, bind to a specific RNA sequences enabling their detection (Tutucci et al. 2018). Unlike MB- or RBLP-based RNA assays, RNA aptamer ‘Spinach’ (known as 24–2 or 24-2 min), and its derivative ‘Spinach2,’ mimic the Green Fluorescent Protein (GFP), thus enabling visualization of targeted RNAs (Paige et al. 2011; Strack et al. 2013; You and Jaffrey 2015). These RNA aptamers bind to the fluorophore DFHBI (3,5-difluoro-4-hydroxybenzylidene imidazolinone) and form an intramolecular G-quadruplex to emit green fluorescence (Huang et al. 2014; Warner et al. 2014). This technology has been successfully used to directly monitor RNAs in bacterial (Paige et al. 2011; Pothoulakis et al. 2014; Zhang et al. 2015), yeast (Guet et al. 2015), and human cells (Paige et al. 2011); and to quantify cellular microRNAs (Huang et al. 2017). More recently, a similar fluorescent RNA aptamer dubbed ‘Pepper’ has also been developed to image RNA in mammalian cells through its binding to the fluorophore ((4-((2-hydroxyethyl)(methyl)amino)-benzylidene)-cyanophenylacetonitrile) (Chen et al. 2019). However, use of fluorescent RNA aptamer-based RNA visualization has so far had little success in plants (Huang et al. 2012; 2017; Bai et al. 2020) although such techniques have attracted a great deal of interest in plant science, particularly in RNA metabolism and mobile RNA signaling (Ehrhardt and Frommer 2012). Unfortunately, the successful establishment of Spinach RNA-mimicking GFP in prokaryotic and eukaryotic cells some ten years ago (Paige et al. 2011) has not led to establish a similar technology in plants. There were many attempts to test this technology, but the only report of using the Spinach aptamer to monitor plant cellular RNAs was unsuccessful (Huang et al. 2017). This has led to the general impression that this technology may not work in plants. In this study, we reevaluated the usefulness of ‘RNA-mimicking-GFP (RMG)’ to assess Spinach-based RMG (S-RMG) in plant cells.

### Materials and methods

#### Construction of vectors

**Fig. 1:**
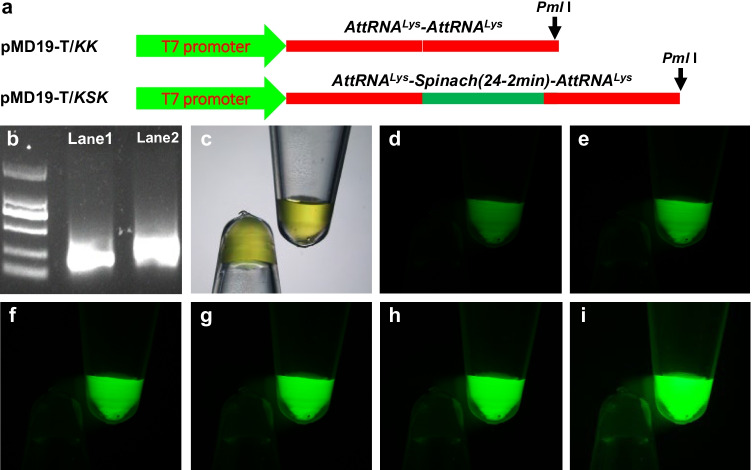
In vitro Spinach RNA fluorescence. **a** Schematic of KK and KSK expression cassettes in pMD19-T. KK (negative control) and KSK were transcribed from *Pml*I-linearized pMD19-T/KK or pMD19-T/KSK under the control of the T7 promoter. Sequences of KK and KSK are included in Data Set S1. **b** 1.5% TAE-agarose gel electrophoresis of KK and KSK RNA transcripts. KK and KSK RNA transcripts were loaded in Lane 1 and Lane 2, respectively. Marker: DM2000. **c**–**i** KK and KSK RNA transcripts in 100 μM DFHBI solution. Photographs were taken under transmitted white light channel (**c**), or under FITC channel at the exposure times of 2 (**d**), 4 (**e**), 6 (**f**), 8 (**g**), 10 (**h**), and 20 (**i**) seconds using a fluorescence stereomicroscope. The concentration of KK and KSK RNA transcripts (**c**–**i**) was 2,915.70 and 2,969.55 ng/μl, respectively

**Fig. 2:**
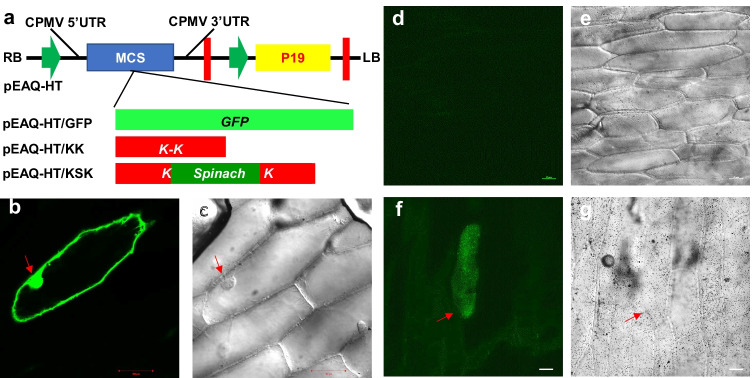
S-RMG in onion epidermal cells. **a** Schematic of GFP, KK, and KSK expression cassettes in pEAQ-HT. GFP, KK, and KSK coding sequences were cloned into the MCS of pEAQ-HT. Green arrows: 35S CaMV promoter sequences. Red vertical lines: CaMV terminator sequences. *MCS* multiple cloning site. CPMV 5′- and 3′-UTR: cowpea mosaic virus 5′ and 3′ untranslated regions which act as translational enhancers. P19: Tombusvirus silencing suppressor protein. **b**–**g** S-RMG in onion epidermal cells. As a control, onion epidermal cells were bombarded with pEAQ-HT/GFP and showed GFP fluorescence at 12 h after bombardment (HAB, **b** and **c**). Onion epidermis was bombarded with pEAQ-HT/KK (**d** and **e**) or pEAQ-HT/KSK (**f** and **g**). Green fluorescence was observed only in onion epidermal cells expressing KSK from with pEAQ-HT/KSK (**f**) at 12 HAB. Photographs were taken under FITC channel (**b**, **d,** and **f**) or through transmitted light (**c**, **e,** and **g**). Bar = 100 μm in **b** and **c**; bar = 50 μm in **d**–**g**. Red arrows indicate cells showing green fluorescence

Original sequences including (i) 73-nucleotides (nt) *AttRNA*^*Lys*^ (K), (ii) 80-nt *Spinach* (S), (iii) 152-nt *AttRNA*^*Lys*^-*AttRNA*^*Lys*^ (KK), (iv) 250-nt *AttRNA*^*Lys*^-*Spinach*-*AttRNA*^*Lys*^ (KSK), (v) 227-nt T7 promoter-KK, and (vi) 374-nt T7 promoter-K-Spinach-K (KSK) are listed in Data Set S1. To obtain double-stranded (ds) KK DNA fragment, a pair of oligonucleotides P001 and P002 (Table S1) was annealed to form a dsDNA molecule. Then, a second pair of oligonucleotides P003 and P004 (Table S1) was also annealed together. The two dsDNA fragments were cloned into the *Mlu*I/*BspE*I sites of the *Potato virus X* (PVX)-based vector (van Wezel et al. 2001) to generate PVX/KK. An *Eag*I site was introduced between the two Ks (Data Set S1). The KK fragment was then amplified from PVX/KK using different sets of primers (Table S1) and subcloned into pMD19-T (TAKARA), or the *Nru*I/*Xho*I sites of pEAQ-HT (Sainsbury et al. 2009) to produce pMD19-T/KK (Fig. 1a), and pEAQ-HT/KK (Fig. 2a), respectively. The T7 promoter sequence and a unique *Pml*I site were introduced to the 5′- or 3′-end of KK in pMD19-T/KK, respectively (Fig. 1a). We cloned the KSK dsDNA fragment which was commercially produced by Invitrogen into the *Age*I/*Sma*I sites of pEAQ-HT and generated pEAQ-HT/KSK (Fig. 2a). The KSK fragment was then amplified from pEAQ-HT/KSK using different sets of primers (Table S1) and subcloned into pMD19-T to produce pMD19-T/KSK (Fig. 1a). In pMD19-T/KK and pMD19-T/KSK, the T7 promoter sequence was incorporated at the 5′-end of KSK while a *Pml*I site was introduced at the 3′-end of KSK (Fig. 1a). The integrity of the sequence insertions in all constructs was confirmed by Sanger sequencing.

#### Preparation of DFHBI solution

Fluorophore DFHBI (3,5-difluoro-4-hydroxybenzylidene imidazolinone) was bought from Lucerna™ company (http://www.lucernatechnologies.com/fluorophores-c17/). DFHBI was dissolved in DMSO to prepare a 40 mM stock solution. It was then diluted with 100 mM HEPES buffer (pH 7.5) to produce a 2 mM DFHBI/5% DMSO working solution (Paige et al. 2011). In this work, the final concentration of DFHBI used to trigger Spinach fluorescence was 100 µM for Spinach RNAs generated by in vitro transcription.

#### Particle bombardment of onion cells and confocal microscopy

Plasmid DNA of pEAQ-HT/KK and pEAQ-HT/KSK was prepared from *Escherichia coli* 2 T1^R^ cells (Thermo Fisher Scientific) using QIAprep Spin Miniprep Kit, and their concentration was adjusted to 1 µg/µl. Gold particles were coated with DNA, and onion epidermal cells were particle-bombarded as described (Ding et al. 2009). Briefly, 1.5 mg of gold microcarriers (1 µm in diameter) were washed with 70% ethanol once and then 100% ethanol twice. After a quick spin, the clean gold microcarriers were collected, air-dried, and resuspended in 50 µl 50% glycerol. Then, 10 µg plasmid DNA, 50 µl 2.5 M CaCl_2_, 20 µl 0.1 M spermidine, and 250 µl 70% ethanol were mixed sequentially and progressively. After a vigorous vortex for 2–3 s, followed by a quick spin, the DNA-coated gold microcarriers were collected, air-dried, and resuspended in 30 µl 100% ethanol. 10 µl DNA-coated gold microcarriers were dropped onto a microparticle carrier disk (Macrocarriers #1,652,335, Bio-Rad), and bombardment was carried out using a PDS-1000/He Biolistic Particle Delivery System (Bio-Rad). After 12-h culture in a hypertonic medium (0.8% Phytagel half-strength Murashige and Skoog (MS) basal medium, 0.256 M (46.67 g/L) sorbitol and 0.256 M (46.67 g/L) mannitol), onion epidermis was immersed into 100 µM DFHBI (3,5-difluoro-4-hydroxybenzylidene imidazolinone) for 30 min, examined and photographed using a Zeiss LSM 710 confocal laser scanning microscope (Ding et al. 2009).

#### In vitro transcription

Production of infectious RNA transcripts was produced by in vitro transcription as described (Hong et al. 2001; Yu et al. 2020a, b). Briefly, pMD19-T/KK and pMD19-T/KSK plasmids were linearized by *Pml*I. The final concentration of purified linear plasmid DNA was 0.25 µg/µl. In vitro transcription was performed using 2.5 µg linear plasmid DNA as template and T7 RNA polymerase (NEB). Purified RNA transcripts were routinely dissolved in 40 µl in RNase-free water. Ten microliters of in vitro RNA transcripts were mixed with 10 µl 200 µM DFHBI, incubated at 75℃ for 5 min, then immediately cooled on ice, and examined and photographed using a Nikon fluorescent stereomicroscope.

## Results and discussion

Prior to delivering the Spinach RNAs into plant cells and tissues, we tested if flanking a plant tRNA at both 5′- and 3′-ends of the Spinach RNA aptamer would affect its binding to DFHBI and fluorescence emission in vitro (Fig. 1). We cloned the *Arabidopsis thaliana* lysine-tRNA (tRNA^Lys^, K) and Spinach (24-2 min) (Paige et al. 2011) in the format of *AttRNA*^*Lys*^-*AttRNA*^*Lys*^ (KK) or *AttRNA*^*Lys*^-*Spinach*(*24-2 min*)-*AttRNA*^*Lys*^ (KSK) into the pMD-19/T vector to generate pMD19-T/KK and pMD19-T/KSK constructs, respectively (Fig. 1a; Data Set S1). The KK and KSK RNA transcription is driven by the T7 promoter in the two expression vectors. Both KK and KSK RNAs produced by in vitro transcription were readily detectable by agarose gel electrophoresis (Fig. 1b). Once mixed with DFHBI, only KSK RNA produced strong GFP-like green fluorescence (Fig. 1c–i). These data indicate that plant tRNA, like its bacterial, yeast, or human counterpart (Paige et al. 2011; Pothoulakis et al. 2014; Zhang et al. 2015; Guet et al. 2015), enables stable and detectable levels of Spinach fluorescence in vitro.

To express Spinach in plant cells, we subcloned *KK* and *KSK* (Fig. 1a; Data Set S1) into pEAQ-HT, a binary vector for efficient gene expression (Sainsbury et al. 2009), and produced pEAQ-HT/KK and pEAQ-HT/KSK (Fig. 2a). Onion epidermal cells without chloroplasts were then bombarded with purified plasmid DNA of pEAQ-HT/KK or pEAQ-HT/KSK. We also bombarded onion tissues with pEAQ-HT/GFP (Sainsbury and Lomonossoff 2008) to express GFP as positive control. GFP fluorescence was readily visible under the confocal microscope in onion epidermal cells 10 h after bombardment (Fig. 2b and c). In striking contrast to KK control (Fig. 2d, e), strong green fluorescence was observed in onion epidermal cells that expressed the KSK RNA in the presence of DFHBI (Fig. 2f, g; Fig. S1; Video S1a-c). Interestingly, we observed that gold particles seem to be also scattered over the surface of the onion cells. However, only these particles that were bombarded into a cell, i.e., inside cell, led to production of Spinach RNAs, and subsequently produced fluorescence in the cytosol in the presence of DFHBI (Fig. 2f, g; Fig. S2). Indeed, no S-RMG signal was found to be associated with gold particles on the cell surface (Fig. S1). Thus, it is possible that insufficient gold particles were delivered inside neighboring cells by particle bombardment to generate detectible RMG fluorescence in these cells. This is also evident in Video S1. Here, at least 4 cells showed obvious RMG signals in the cytosol, and gold particles on the cell surface did not produce fluorescence. We also noticed that the distribution of the RMG signal differed from GFP fluorescence (Fig. 2b, f; Fig. S1; Fig. S2; Video S1). However, it is worthwhile noting that GFP is a protein while Spinach is an RNA. Proteins and RNAs are usually distributed in different fashions in cells. Nevertheless, the S-RMG signal distribution in onion cells is similar to those seen in mammalian cells (Paige et al. 2011). Thus, in contrast to a previous report (Huang et al. 2017), we have demonstrated genuine S-RMG visualization in onion cells, clearly demonstrating that the Spinach-based RMG can work in plant cells.

In conclusion, the Spinach RNA aptamer can mimic GFP in plant cells. It should be noted that because the Spinach RNA aptamer can mimic GFP in plant cells, it does not affect the location of Spinach RNA aptamer and which cells are involved in. So, will it still work on leaves with chloroplast? In theory, S-RMG should work on leaves with chloroplasts. However, background chloroplast autofluorescence can limit the sensitivity of S-RMG in leaf tissues (Yu et al. 2020b). Nevertheless, our work also indicates that the full potential of a Spinach-based RMG technology in plant RNA visualization is worth further investigation. Moreover, since the development of Spinach (Paige et al. 2011), several new aptamers such as Spinach2 (Strack et al. 2013), Baby Spinach (Huang et al. 2014), iSpinach (Autour et al. 2016), Pandan (Aw et al. 2016), Broccoli (Filonov et al. 2014), RNA-Mango (Dolgosheina et al. 2014), Corn-DFHO (Warner et al. 2017), and Pepper (Chen et al. 2019) have been discovered and used for RNA visualization. Spinach2, Baby Spinach, and iSpinach also use DFHBI as a fluorophore, and these derivatives are superior to Spinach in terms of fluorescence intensity and/or the light quenching properties (Chen et al. 2019; Filonov et al. 2014; Warner et al. 2014). In addition, red Broccoli may also be useful due to the spectral shift, which may help overcome background fluorescence. Because the 49-nt Broccoli is thought to be a better folding aptamer than the 98-nt Spinach and it yet has many of the same structural features of Spinach and binds the same fluorophore (Paige et al. 2011; Filonov et al. 2014), Broccoli is also perceived to be better than Spinach in terms of length of aptamer sequences, brightness of fluorescence, and fluorophore affinity (Filonov et al. 2019). These newly developed aptamers do not require tRNA scaffolds for protection, and they can still stably bind to RNA and stimulate fluorescence (Chen et al. 2019; Filonov et al. 2014). Thus, these newer RNA aptamers offer more options for RNA visualization *in planta*. Indeed, a series of fluorescent aptamers were created based on the modified three-way junction scaffold and the optimized Broccoli has been elegantly used to visualize RNA in plants (Bai et al. 2020). Our data shows for the first time that the Spinach-based RMG also works in plant cells.

## Supplementary Information

Below is the link to the electronic supplementary material.

Supplementary file1 (PDF 520 KB)

Supplementary file2 (XLSX 12 KB)

Supplementary file3 (PPTX 4828 KB)
