# Supplementary material for: Spinach-based RNA mimicking GFP in plant cells
Source: Funct Integr Genomics. 2022 Mar 10;22(3):423–8. doi: 10.1007/s10142-022-00835-x (PMC9197860; doi:10.1007/s10142-022-00835-x)
Supplement: Supplementary file 1 — Supplementary file1 (PDF 520 KB) [file 10142_2022_835_MOESM1_ESM.pdf]

SHORT COMMUNICATION

SUPPLEMENTARY INFORMATION

Spinach-based RNA mimicking GFP in plant cells

Zhiming Yu[1,*], Yue Wang[1], Fengling Mei[1], Haiting Yan[1], Zhenhui Jin[1], Pengcheng Zhang[1,2], Xian Zhang[1], Mahmut Tör[2], Stephen Jackson[3], Nongnong Shi[1], Yiguo Hong[1,2,3,*]

[1]Research Centre for Plant RNA Signaling, College of Life and Environmental Sciences, Hangzhou Normal University, Hangzhou 311121, China

[2]School of Science and the Environment, University of Worcester, Worcester WR2 6AJ, UK

[3]School of Life Sciences, University of Warwick, Coventry CV4 7AL, UK

[*]Correspondence:
Yiguo Hong
yiguo.hong@hznu.edu.cn; yiguo.hong@warwick.ac.uk; y.hong@worc.ac.uk

Co-correspondence:
Zhiming Yu
yuzhiming@hznu.edu.cn

Running Title: S-RMG in plants

Data Set S1. Sequence information

The original sequences used for all constructs (Fig 1a; Fig. 2a) are shown below.

(i) *AttRNA*^{Lys} (abbreviated to K, 73 nt):

5’-GCCCGTCTAG-CTCAGTTGGT-AGAGCGCAAG-GCTCTTAACC-TTGTGGTCGT-GGGTTCGAGC-CCCACGGTGG-GCG-3’

(ii) *Spinach* sequence (abbreviated to S, 80 nt):

5’-GACGCGACCG-AAATGGTGAA-GGACGGGTCC-AGTGCTTCGG-CACTGTTGAG-TAGAGTGTGA-GCTCCGTAAC-TGGTCGCGTC-3’

(iii) *AttRNA*^{Lys}-*AttRNA*^{Lys} (abbreviated to KK) sequence (152 nt):

5’-GCCCGTCTAG-CTCAGTTGGT-AGAGCGCAAG-GCTCTTAACC-TTGTGGTCGT-GGGTTCGAGC-CCCACGGTGG-GCG**cggccg**G-CCCGTCTAGC-TCAGTTGGTA-GAGCGCAAGG-CTCTTAACCT-TGTGGTCGTG-GGTTCGAGCC-CCACGGTGGG-CG-3’ (The underline sequence is the *Eag* I site.)

(iv) *AttRNA*^{Lys}-*Spinach*-*AttRNA*^{Lys} (abbreviated to KSK) sequence (250 nt):

5’-accggt**GCCC-GTCTAGCTCA-GTTGGTAGAG-CGCAAGGCTC-TTAACCTTGT-GGTCGTGGGT-TCGAGCCCCA-CGGTGGGCG**a agctt**GACGC-GACCGAAATG-GTGAAGGACG-GGTCCAGTGC-TTCGGCACTG-TTGAGTAGAG-TGTGAGCTCC-GTAACTGGTC-GCGTC**gcatg-c**GCCCGTCTA-GCTCAGTTGG-TAGAGCGCAA-GGCTCTTAAC-CTTGTGGTCG-TGGGTTCGAG-CCCCACGGTG-GGCG**cccggg-3’ (The underline sequence from 5’ to 3’ is *Age* I, *Hind* III, *Sph* I and *Sma* I (*Xma* I) site, respectively. The green sequence is *Spinach1*. The bold sequence on each side of *Spinach* is *AttRNA*^{Lys}.)

(v) T7/KK sequence (227 nt):

5’-taatacgactcactatagggTCACCACCAC-GGAATCGATacgcgt**GCCCG-TCTAGCTCAG-TTGGTAGAGC-GCAAGGCTCT-TAACCTTGTG-GTCGTGGGTT-CGAGCCCCAC-GGTGGGCG**cggccg**GCCCGT-CTAGCTCAGT-TGGTAGAGCG-CAAGGCTCTT-AACCTTGTGG-TCGTGGGTTC-GAGCCCCACG-GTGGGCGTCC-GGATGATATC-GTCGACCGCC-G**cacgtg-3’ (The sequence in the box is the T7 promoter. The underline sequence from 5’ to 3’ is *Mlu* I, *Eag* I, and *Pml* I site respectively.)

(vi) T7/K-Spinach-K sequence (KSK, 374 nt)

5’-taatacgactcactatagggTCACCACCAC-GGAATCGATacgcgtATATT-CTGCCCAAAT-TCGCGaccggt**GCCCGTCTA-GCTCAGTTGG-TAGAGCGCAA-GGCTCTTAAC-CTTGTGGTCG-TGGGTTCGAG-CCCCACGGTG-GGCG**aagctt**GACGCGACCG-AAATGGTGAA-GGACGGGTCC-AGTGCTTCGG-CACTGTTGAG-TAGAGTGTGA-GCTCCGTAAC-TGGTCGCGTC-**gcatgc**GCCC-GTCTAGCTCA-GTTGGTAGAG-CGCAAGGCTC-TTAACCTTGT-GGTCGTGGGT-TCGAGCCCCA-CGGTGGGCG**cccgggCATCA-CCATCACCAT-CACTACGGCC-GTGATCCGGA-TGATATCGTC-GACCGCCGca cgtg-3’ (The sequence in the box is the T7 promoter. The underline sequence from 5’ to 3’ is *Mlu* I, Age I, *Hind* III, *Sph* I, *Sma* I (*Xma* I) and *Pml* I site respectively. The green sequence is Spinach. The bolded sequence on each side of *Spinach* is *AttRNA*^{Lys}.)

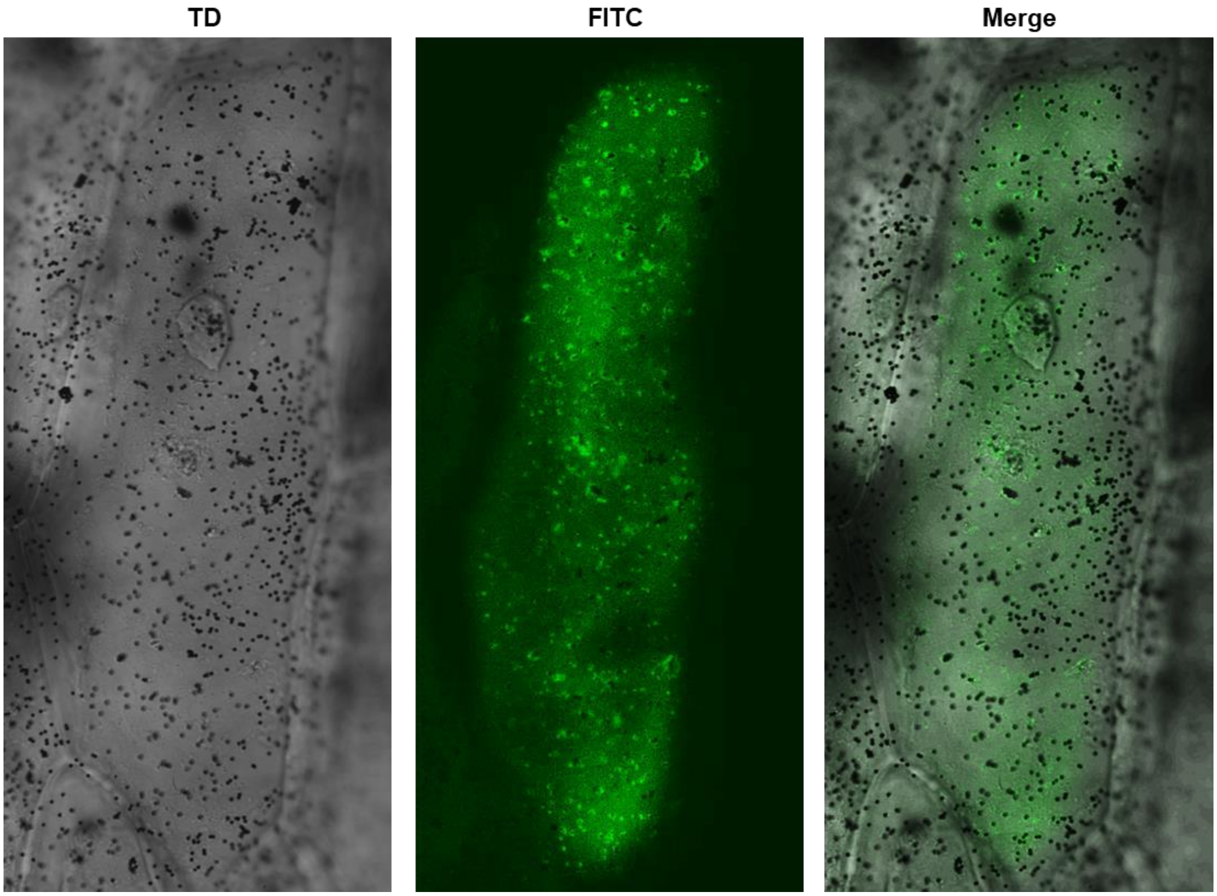

FIGURE S1. Spinach-based RMG in onion epidermal cells. The onion cell in Fig. 2f was enlarged to show RMG in detail. Under transmitted white light (TD), numerous gold particles coated with pEAQ-HT/KSK were visible as “dark” dots (Left panel). Strong green fluorescence was observed under the FITC filter (middle panel). The merged image (right) clearly shows green fluorescence around the “dark” gold particle and throughout the cytoplasm of this onion epidermal cell. Photograph was taken as described in the legend of Fig. 2.

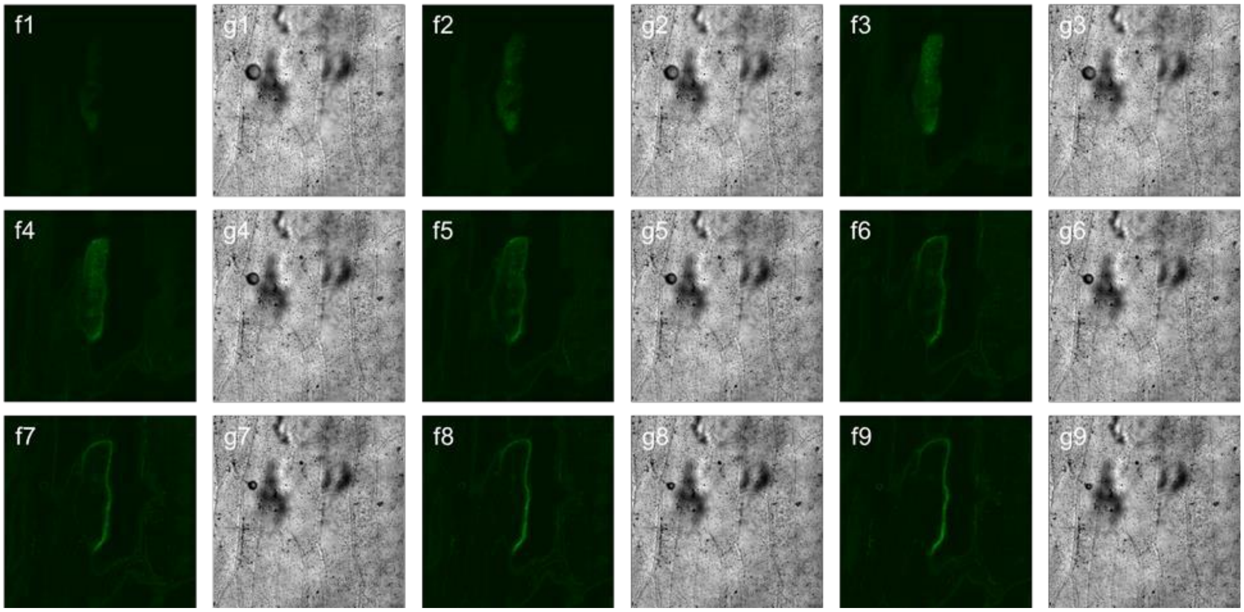

FIGURE S2. S-RMG within bombarded onion cells. S-RMG fluorescent and TD images of different layers of laser scanning under confocal microscopy. Images f1-f9, or g1-g9 have been used to produce Fig. 2f and Fig. 2g, respectively. Gold particles were scattered over surface of all onion cells. However, only these particles that were bombarded inside cell resulted in production of Spinach RNAs, and subsequently producing green fluorescence in the presence of DFHBI in cytosol. No S-RMG signal was found to be associated with gold particles on the cell surface. Together with results presented in Fig. 2f, g; Fig. S1 and Video S1, these images shown here clearly demonstrate the S-RMG signal is within the cell, and that gold particles on the cell surface does not produce fluorescence.
