# Supplementary material for: Spinach-based RNA mimicking GFP in plant cells
Source: Funct Integr Genomics. 2022 Mar 10;22(3):423–8. doi: 10.1007/s10142-022-00835-x (PMC9197860; doi:10.1007/s10142-022-00835-x)
Supplement: Supplementary file 3 — Supplementary file3 (PPTX 4828 KB) [file 10142_2022_835_MOESM3_ESM.pptx]

## Slide 1
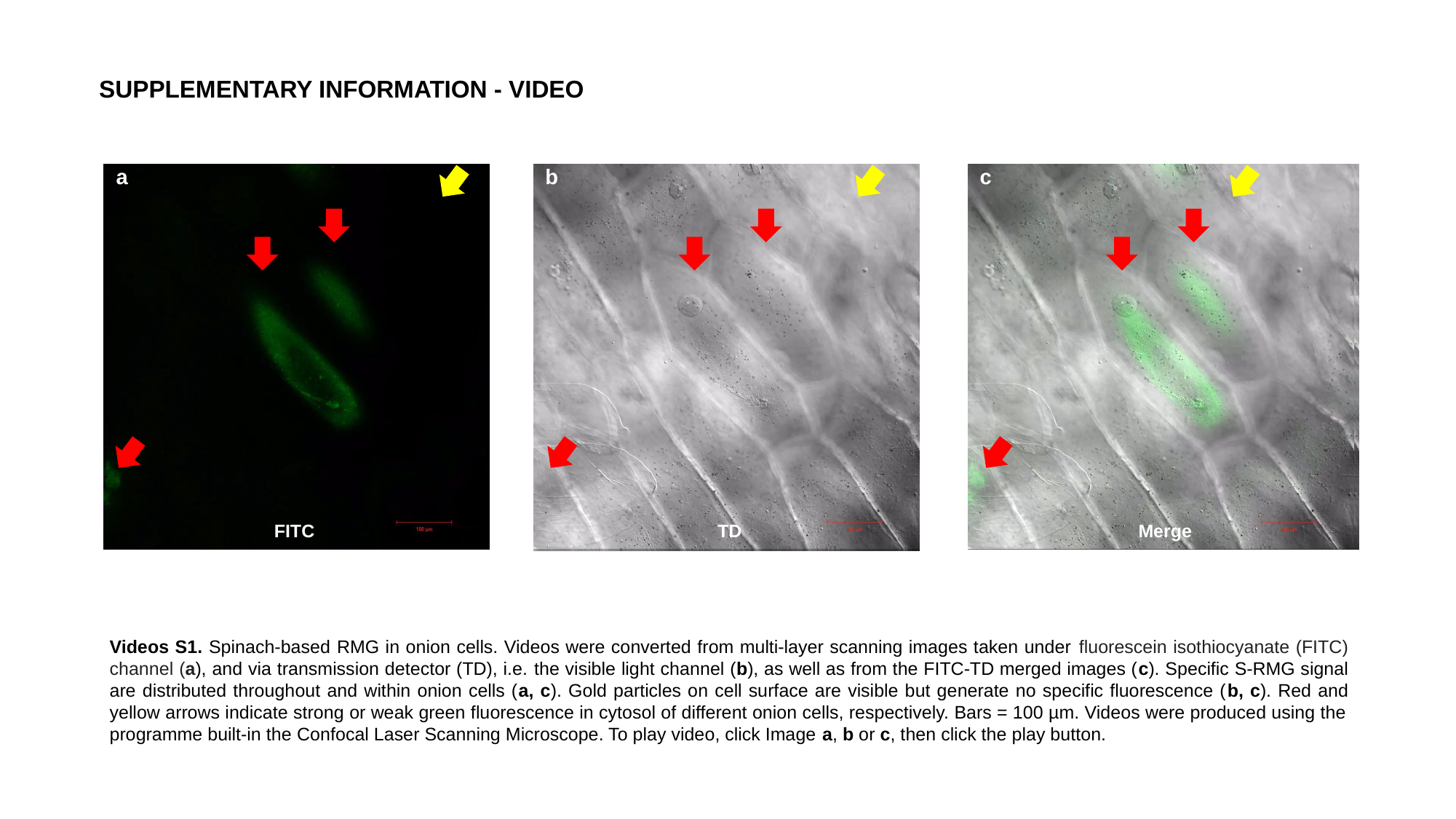

SUPPLEMENTARY INFORMATION - VIDEO
a
b
c
FITC
TD
Merge
Videos S1. Spinach-based RMG in onion cells. Videos were converted from multi-layer scanning images taken under fluorescein isothiocyanate (FITC) channel (a), and via transmission detector (TD), i.e. the visible light channel (b), as well as from the FITC-TD merged images (c). Specific S-RMG signal are distributed throughout and within onion cells (a, c). Gold particles on cell surface are visible but generate no specific fluorescence (b, c). Red and yellow arrows indicate strong or weak green fluorescence in cytosol of different onion cells, respectively. Bars = 100 µm. Videos were produced using the programme built-in the Confocal Laser Scanning Microscope. To play video, click Image a, b or c, then click the play button.
